# *Achyranthes bidentata polysaccharides* attenuate hypoxic renal injury by reducing neutrophil extracellular traps and suppressing the NLRP3/ASC/caspase-1 pathway: a preliminary study

**DOI:** 10.3389/fphar.2025.1606851

**Published:** 2025-10-31

**Authors:** Kai Li, JingWei Fang, GuangMin Xie, FangNing Wei, JunYing Lv

**Affiliations:** ^1^ The Second Clinical Medical College, Guangzhou University of Chinese Medicine, Guangzhou, China; ^2^ Department of Traditional Chinese Medicine, The First Affiliated Hospital of Guangxi Medical University, Nanning, China; ^3^ Department of Nephrology, The Second Clinical Medical College, Guangzhou University of Chinese Medicine, Guangzhou, China

**Keywords:** *Achyranthes bidentata* polysaccharides, neutrophil extracellular traps, oxidative stress, hypoxic renal injury, acute kidney injury

## Abstract

**Aim:**

The aim of this study was to investigate whether *Achyranthes bidentata* polysaccharides (ABPSs) alleviate hypoxic renal injury (HRI) and the possible mechanism.

**Methods:**

The HRI rat model was established using a hypobaric hypoxia chamber. Rats were divided into a control group, a hypoxia group, low-dose ABPS (ABPL) group, a high-dose ABPS (ABPH) group, a DNase I-positive control group, and an NLRP3 agonist nigericin sodium salt (NSS) group. Blood serum components relevant to neutrophil extracellular traps (NETs), including cell-free DNA (cf-DNA), myeloperoxidase-DNA (MPO-DNA), neutrophil elastase-DNA (NE-DNA), citrullinated histone 3 (cit-H3), blood urea nitrogen (BUN), serum creatinine (Scr), cystatin C (CysC), neutrophil gelatinase-associated lipocalin (NGAL), and kidney injury molecule-1 (KIM-1), were analyzed. NOD-like receptor protein 3 (NLRP3) pathway proteins, MPO, NE, cit-H3, and reactive oxygen species (ROS) in renal tissues were analyzed by multiplex fluorescence immunohistochemistry. MPO and cit-H3 in renal tissues were analyzed via Western blot. Oxidative stress markers such as malondialdehyde (MDA) and superoxide dismutase (SOD) were analyzed using the thiobarbituric acid (TBA) assay.

**Results:**

The results demonstrated that ABPSs exerted protective effects against hypoxic renal injury. First, ABPSs significantly reduced the levels of cf-DNA, MPO-DNA, and cit-H3, with efficacy comparable to that of DNase I. Second, ABPSs markedly suppressed the activation of the NLRP3 inflammasome pathway by degrading NETs, as evidenced by reduced protein expression of NLRP3, apoptosis-associated speck-like protein containing a CARD (ASC), and caspase-1, accompanied by significant decreases in interleukin 1β (IL-1β) and IL-18. Furthermore, ABPSs effectively alleviated oxidative stress by reducing MDA, enhancing SOD activity, and attenuating ROS. Finally, these molecular and cellular improvements translated into functional recovery as high-dose ABPS treatment restored renal function to near-normal levels, including a 58.1% reduction in BUN, a 34.5% reduction in Scr, a 23.6% reduction in NGAL, a 29.6% reduction in KIM-1, and a 32.2% reduction in CysC. Hematoxylin and eosin (H&E) and periodic acid–Schiff (PAS) staining and quantitative scoring analysis of kidney injury revealed severe tubular necrosis and glomerular damage in rats in the hypoxia group, which were significantly attenuated in both the ABPL and ABPH groups (*p* < 0.05).

**Conclusion:**

ABPS mitigates hypoxic renal injury by reducing NETs and synergistically regulating oxidative stress. ABPS shows potential as a multi-target, low-toxicity candidate for renal protection.

## 1 Introduction

Hypoxic renal injury (HRI), a critical pathological subtype of acute kidney injury (AKI), accounts for 35%–55% of AKI-related hospitalizations, particularly in patients undergoing cardiac surgery, sepsis, or nephrotoxic drug exposure (Hoste et al., 2018). Globally, AKI affects approximately 2,130 individuals per million annually, with in-hospital mortality rates as high as 23.9%. Notably, 30% of survivors develop chronic kidney disease, significantly elevating cardiovascular risks and socioeconomic burdens (Susantitaphong et al., 2013). Hypoxia-induced renal damage is driven by mitochondrial dysfunction, oxidative stress, and sterile inflammatory responses, leading to tubular epithelial cell necrosis and microcirculatory impairment. However, therapeutic strategies targeting these multifactorial mechanisms remain limited (Bonventre and Yang, 2011).

Neutrophil extracellular traps (NETs), composed of cell-free DNA (cf-DNA), citrullinated histone 3 (cit-H3), myeloperoxidase (MPO), and neutrophil elastase (NE), exacerbate tissue damage by releasing damage-associated molecular patterns (DAMPs) that activate innate immunity (Gupta and Kaplan, 2016). In ischemic renal injury, NET-derived components such as cf-DNA and high-mobility group box 1 initiate NOD-like receptor family pyrin domain containing 3 (NLRP3) inflammasome assembly via Toll-like receptor 4 (TLR4) signaling (Nakazawa et al., 2017; Huang et al., 2015). Clinical studies further demonstrate a strong correlation between serum myeloperoxidase-DNA (MPO-DNA) in AKI patients, highlighting the therapeutic potential of targeting the NETs (Desai et al., 2016).

*Achyranthes bidentata* polysaccharides (ABPSs), the primary bioactive constituents of the traditional botanical drug *Achyranthes bidentata (Amaranthaceae)*, exhibit anti-inflammatory, antioxidant, and immunomodulatory properties (Chai et al., 2024). ABPSs inhibited lipopolysaccharide (LPS)-induced NLRP3 and cleaved caspase 1 expression (Wang et al., 2021). ABPSs attenuate LPS-induced AKI by inhibiting reactive oxygen species (ROS) and apoptosis via an estrogen-like pathway (Wang et al., 2020). The present study aimed to investigate the mechanism by which ABPSs attenuate hypoxic renal injury.

## 2 Materials and methods

### 2.1 Hypobaric hypoxia-induced renal injury model

A hypobaric hypoxia-induced acute renal injury model was established in Sprague–Dawley male rats (120–150 g) following protocols described by Wei et al. (2023). Rats were housed in a hypoxic chamber maintained at 23 °C ± 2 °C with 50%–60% relative humidity and were allowed free access to food and water. The chamber was opened for 30 min daily for drug administration and replenishment of supplies. Control rats were maintained under normoxic conditions.

A total of 128 rats were randomly assigned to 16 experimental groups (n = 8 per group), structured according to the following three experimental protocols.

Role of NETs in hypobaric hypoxia-induced AKI: control group and hypobaric hypoxia-induced renal injury model (hypoxia) group.

Effects of different doses of ABPSs on renal function: control group, hypoxia group, ABPS low-dose intervention (ABPL) group, ABPS high-dose intervention (ABPH) group, and dexamethasone (DEX) group.

**FIGURE 1:**
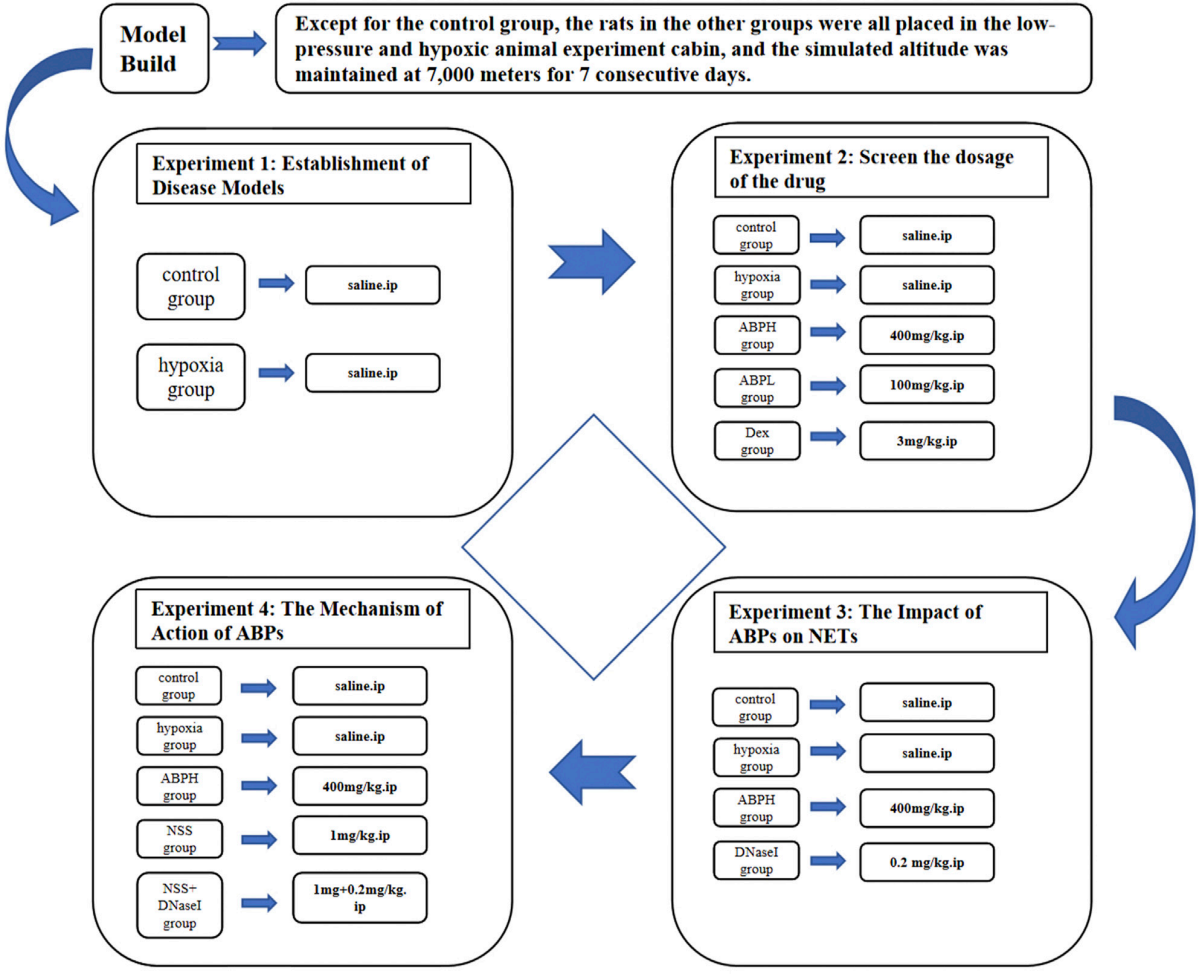
Experimental design of the different groups and their respective study parameters.

Impacts of ABPSs on NETs and underlying mechanisms:

1) NET modulation: control group, hypoxia group, ABPH group, and deoxyribonuclease I (DNase I) group.

2) Mechanism of ABPSs in alleviating hypobaric hypoxia-induced AKI: control group, hypoxia group, ABPH group, NLRP3 agonist nigericin sodium salt (NSS) group, and NSS + DNase I group (Figure 1).

Due to budgetary constraints, a randomly selected subset of six animals from each group was used for Western blot, ROS, immunohistochemistry, and multiplex immunofluorescence analyses of NETs. The selection was performed using a computer-generated random number table to ensure representativeness and prevent selection bias.

Drug administration: Dexamethasone (3.0 mg/kg, i.p.) was purchased from Tianjin Jin Yao Pharmaceutical Co., Ltd., China. DNase I (0.2 mg/kg, i.p., HY-108882C) and NSS (1 mg/kg, HY-100381) were obtained from MedChemExpress, United States. ABPS (100 or 400 mg/kg, HY-100381) was purchased from Shanghai Ronghe Pharmaceutical Technology Development Co., Ltd. The DNase I and NSS doses were based on previous studies (Chen et al., 2023; Shan et al., 2021). The ABPS dose was 100 mg/kg in the ABPL group and 400 mg/kg in the ABPH group, and the control group was given the same volume of normal saline via intravenous injection at the corresponding administration time points according to the previous studies (Lin et al., 2010; Fu et al., 2022a; Lang et al., 2019). All the drugs were administered daily for 7 days after hypoxia induction (Figure 1).

### 2.2 Preparation and characterization of ABPS

ABPS, along with its homogeneity and molecular weight data, was obtained from Shanghai Ronghe Pharmaceutical Technology Development Co., Ltd. In brief, dried roots of *Achyranthes bidentata* were sectioned and extracted with distilled water at room temperature overnight. The aqueous extract was filtered and then concentrated under reduced pressure, and the resulting supernatant was precipitated with acetone at 4 °C overnight. Crude polysaccharides were obtained following deproteinization, filtration, and concentration. Furthermore, purification was achieved by sequential chromatography on DEAE-Cellulose 52 and Sephadex G-50 columns, yielding ABPS with an extraction rate of 1.5% (Fan et al., 2021).

**FIGURE 2:**
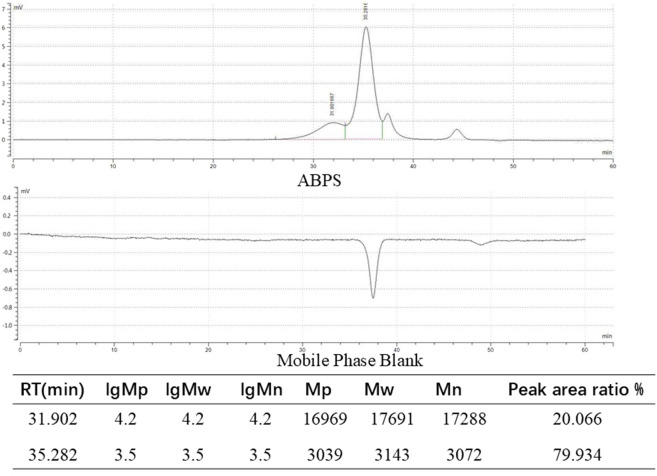
ABPSs were analyzed by high-performance gel permeation chromatography (HPGPC).

The homogeneity and molecular weight of ABPS were analyzed by high-performance gel permeation chromatography (HPGPC) using a Shimadzu LC-10A System. Monosaccharide composition was determined by ion chromatography using a Thermo Fisher ICS 5000 instrument. ABPS was found to be composed of glucose, galactose, arabinose, and rhamnose, and the HPGPC results are shown in Figure 2.

### 2.3 Renal function and injury marker detection

Serum was prepared from tail vein blood collected from each group of rats for subsequent testing. Blood urea nitrogen (BUN, Shanghai Jining Biotechnology, Shanghai, JN5173, China), serum creatinine (Scr, Nanjing Jiancheng Biological Engineering Institute, Nanjing, C011-2-1, China), cystatin C (CysC, Wuhan Yunclone Technology Co., Ltd., Wuhan, SEA896Ra, China), neutrophil gelatinase-associated lipocalin (NGAL, Nanjing Jiancheng Biological Engineering Institute, Nanjing, H392-1-2, China), and kidney injury molecule-1 (KIM-1, Wuhan Yunklon Technology Co., Ltd., Wuhan, SEA785Ra, China) were quantified using commercial ELISA kits, according to the manufacturer’s instructions. The serum samples for NGAL testing were diluted to 1:10. The serum samples for BUN and CysC testing were diluted to 1:5. The serum samples for KIM-1 and Scr testing required no dilution.

### 2.4 Quantitative analysis of NETs

The cf-DNA, MPO-DNA, and NE-DNA levels were analyzed using ELISA with relevant assay kits (MPO-DNA, MM-71053R2; cf-DNA, MM-72009R2; NE-DNA, MM-72016R2, Meimian, Jiangsu, China). The serum samples for MPO-DNA and NE-DNA testing were diluted to 1:5. The serum samples for cf-DNA testing required no dilution.

NET visualization: Renal sections were stained with 4′,6-diamidino-2-phenylindole (DAPI C1002, Beyotime Biotechnology, China), anti-MPO (Abcam, ab208670, United Kingdom), anti-NE (Abcam, ab314936, United Kingdom), and cit-H3 (Abcam, ab281584, United Kingdom), according to the previous study (Chen et al., 2021). NET deposition was visualized using a 3DHISTECH Digital Slice Scanning System (Pannoramic, 3DHISTECH Ltd., Hungary).

The expressions of MPO and cit-H3 proteins in kidney tissue samples were analyzed. Western blotting was performed using anti-MPO (1:1000, Abcam, United Kingdom) and anti-cit-H3 (1:1000, Abcam, United Kingdom) antibodies. Band intensities were quantified using ImageLab software (Bio-Rad, Hercules, CA, United States).

### 2.5 Oxidative stress and inflammation assessment

Oxidative stress markers: Malondialdehyde (MDA) and superoxide dismutase (SOD) levels in renal tissues were analyzed using the thiobarbituric acid assay (MDA, Nanjing Jiancheng Biological Engineering Institute, Nanjing, A003-1, China; SOD, Beyotime Biotechnology, S0101S, China) at 532 nm and the xanthine oxidase method to measure superoxide anion inhibition, respectively. The homogenate samples for MDA and SOD testing were diluted to 1:10 and 1:5, respectively. ROS: Renal tissue sections were incubated with 5 μM dihydroethidium (S0064S, Beyotime Biotechnology, China) at 37 °C, followed by nuclear counterstaining with DAPI. Renal tissue supernatants were analyzed for inflammatory cytokines using ELISA kits for IL-1β, IL-18, and TNF-α (IL-1β, F2923A; TNF-α, F3056A; and IL-18, F3070A, Shanghai FANKEW, China). The homogenate samples for TNF-α and IL-18 testing were diluted to 1:10, and those for IL-1β testing were diluted to 1:5.

### 2.6 NLRP3 inflammasome pathway analysis

Protein expressions of NLRP3, ASC, and caspase-1 were analyzed by Western blot using anti-NLRP3 (1:500, Jiangsu Pico Biological Research Center Co., Ltd., Jiangsu, DF7438, China, Affinity Biosciences), anti-ASC (1:500, Jiangsu Pico Biological Research Center Co., Ltd., Jiangsu, DF6304, China, Affinity Biosciences), anti-caspase-1 (1:500, AF5418, Affinity Biosciences), and anti-cleaved-caspase 1 (1:500, AF4005, Affinity Biosciences) antibodies.

### 2.7 Histopathological analysis

Paraffin-embedded renal sections were stained with hematoxylin and eosin (H&E) to evaluate inflammatory infiltration and periodic acid–Schiff (PAS) to assess tubular basement membrane damage. Images were captured using a light microscope. Quantitative scoring analysis of kidney histopathological injury was also performed.

### 2.8 Statistical analysis

Data are expressed as the mean ± standard deviation. Intergroup differences were analyzed using one-way ANOVA, followed by Tukey’s multiple comparison, and the unpaired t-test was used to compare the data between the two groups in SPSS 27.0 (IBM, United States). A *p*-value <0.05 was considered statistically significant.

## 3 Results

### 3.1 NETs involved in hypobaric hypoxia-induced AKI

**FIGURE 3:**
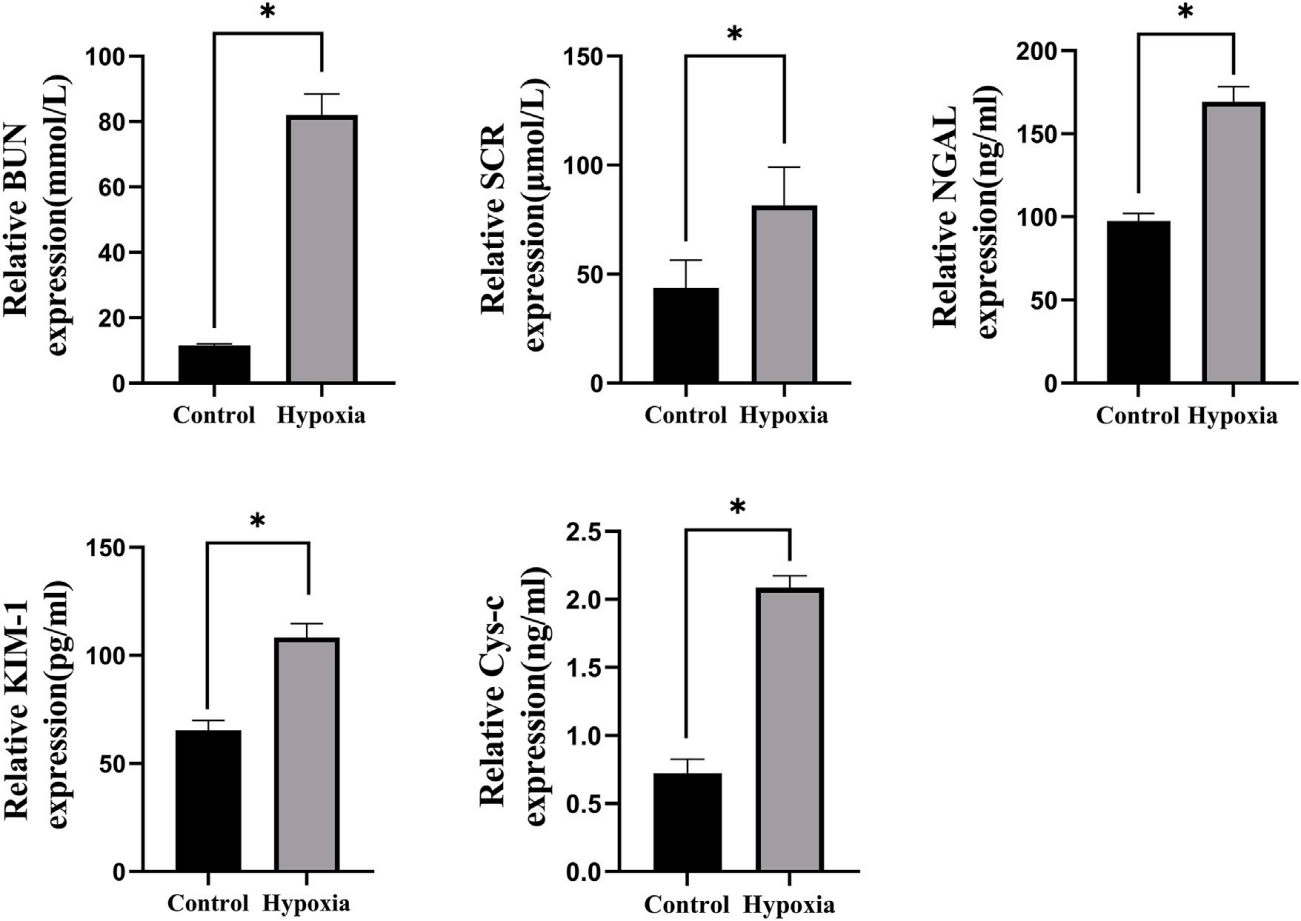
BUN, Scr, NGAL, KIM-1, and CysC levels, *p < 0.001.

The hypobaric hypoxia-induced acute renal injury model was successfully established in rats, as demonstrated by significant increases in renal dysfunction markers. BUN levels in the hypoxia group were 613.4% higher than those in the control group (82.05 ± 6.41 mmol/L vs. 11.55 ± 0.45 mmol/L, *p* < 0.001). Similarly, in the hypoxia group, Scr levels increased by 86.5% (81.52 ± 17.6 μmol/L vs. 43.71 ± 12.74 μmol/L, *p < 0.001*), NGAL levels increased by 73.3% (169.20 ± 9.18 ng/mL vs. 97.61 ± 4.43 ng/mL, *p* < 0.05), KIM-1 levels increased by 65.6% (108.32 ± 6.32 pg/mL vs. 65.4 ± 4.53 pg/mL, *p < 0.001*), and CysC levels increased by 190.2% (2.09 ± 0.08 ng/mL vs. 0.72 ± 0.1 ng/mL, *p* < 0.001) compared to those in the control group (Figure 3).

**FIGURE 4:**
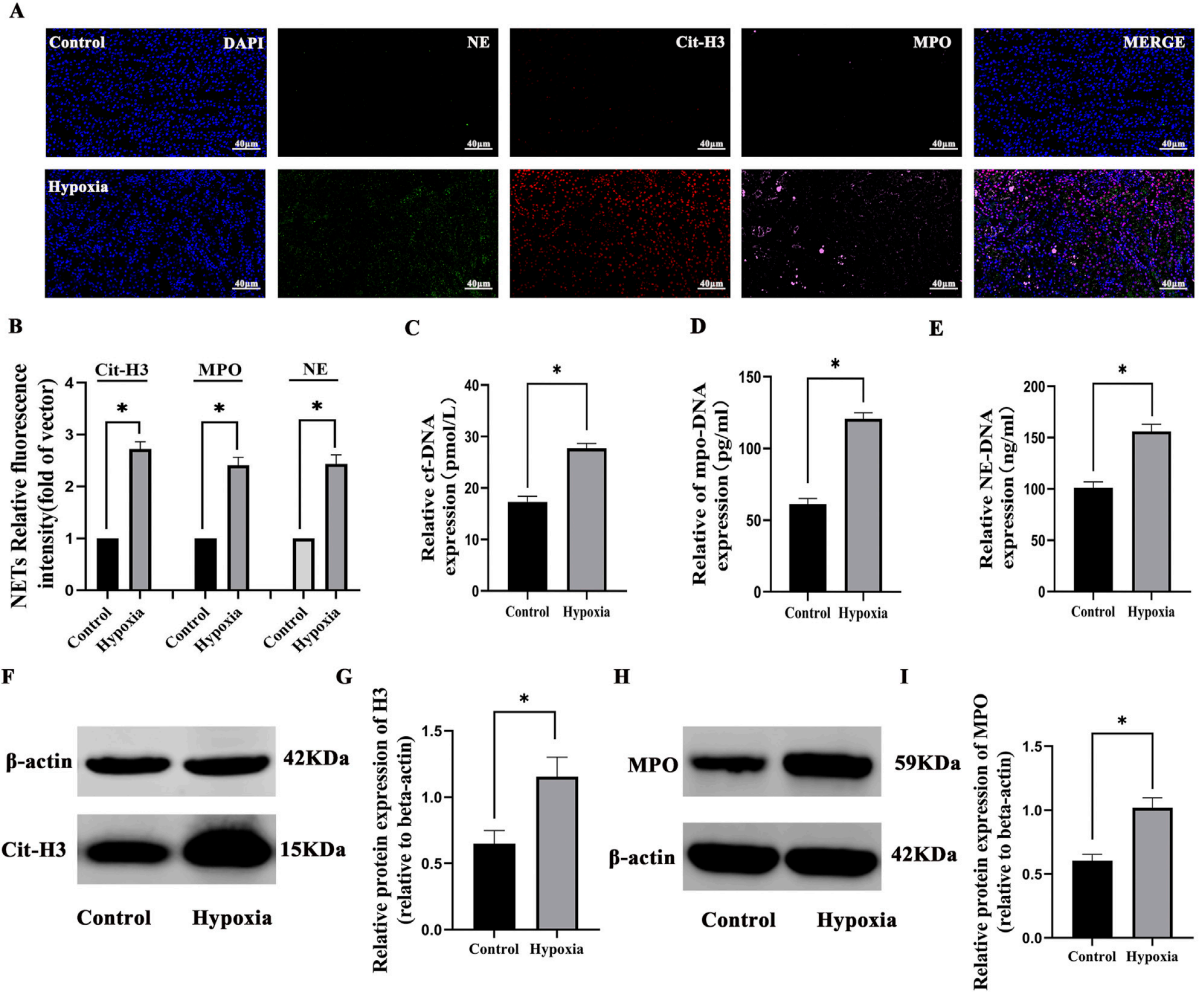
Formation of neutrophil NETs in hypobaric hypoxia-induced AKI: **(A)** immunofluorescence staining of neutrophil elastase (NE, green), myeloperoxidase (MPO, pink), and citrullinated histone H3 (cit-H3, red) in renal tissues (×400). Nuclear DNA was counterstained with DAPI (blue). **(B)** Quantitative analysis of NETs. **(C–E)** The cf-DNA, MPO-DNA, and NE-DNA levels significantly increased compared to those in the control group. **(F–I)** Western blot analysis revealed that Cit-H3 and MPO protein expressions significantly increased in the hypoxia group compared to those in the control group. *P < 0.05.

Concurrently, NETs were significantly elevated under hypoxic conditions compared to the control group through multiplex fluorescence immunohistochemistry and semi-quantitative analysis (*p* < 0.05) (Figures 4A,B). Cf-DNA levels increased by 60.2% (27.76 ± 0.93 pmol/L vs. 17.32 ± 1.08 pmol/L, *p* < 0.001), MPO-DNA by 97.5% (120.8 ± 4.17 pg/mL vs. 61.17 ± 3.92 pg/mL, *p* < 0.001), NE-DNA by 54.3% (156.17 ± 6.93 ng/mL vs. 101.19 ± 5.68 ng/mL, *p* < 0.001), the WB levels of MPO by 68.3% (1.01 ± 0.07 vs. 0. 60 ± 0. 04, *p* < 0.05), and cit-H3 by 79.7% (1.15 ± 0.14 vs. 0.64 ± 0.09, *p* < 0.05). These results confirmed that hypoxic injury robustly activates NET formation, which correlates with renal functional impairment (Figures 4C–E). Finally, the WB results of NET-related proteins cit-H3 and MPO also confirmed that NETs were significantly increased after hypoxia compared with the control group (*p* < 0.05) (Figures 4F–I).

### 3.2 Comparative effects of *ABPS* interventions on renal function

**FIGURE 5:**
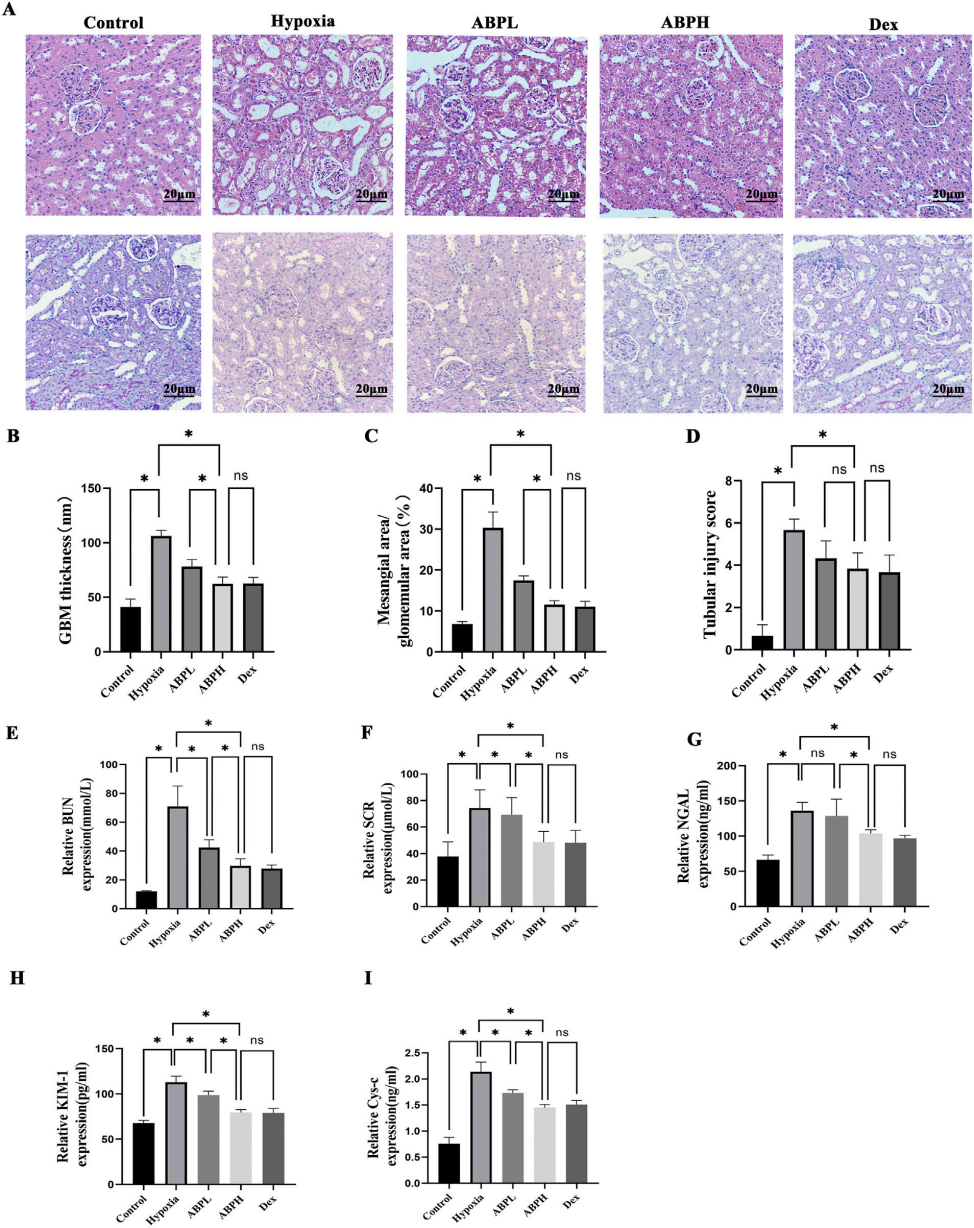
**(A)** H&E and PAS staining (×200) revealed renal histopathological changes. Renal tissues from the control group exhibited no structural damage. In contrast, the hypoxia group showed severe renal injury, including cortical tubular epithelial cell swelling, cytoplasmic vacuolization and pallor, extensive tubular dilation, flattened sloughed tubular epithelial cells, and interstitial lymphocyte infiltration. Both ABPL (low-dose *Achyranthes bidentata* polysaccharide) and ABPH treatments significantly alleviated these pathological alterations, with ABPH demonstrating a more pronounced protective effect compared to the DEX-positive control group. **(B–D)** Quantitative scoring analysis of kidney injury. *p < 0.05. ABPL, ABPH, and DEX treatments substantially reduced mesangial hyperplasia, with ABPH and DEX showing the most significant attenuation of glomerular damage. **(E–I)** BUN, Scr, NGAL, KIM-1, and CysC levels, *p < 0.05.

The renoprotective effects of *ABPS* interventions were evaluated across low- and high-dosing regimens. H&E and PAS staining and quantitative scoring analysis of kidney injury revealed severe tubular necrosis and glomerular damage in rats of the hypoxia group, which were significantly attenuated in both ABPL and ABPH groups (*p* < 0.05). ABPH exhibited near-normal renal architecture with minimal inflammatory infiltration, underscoring its dose-dependent therapeutic superiority (*p* < 0.05) (Figures 5A–D).

Dose-dependent renoprotection by ABPSs in hypoxic renal injury: Low-dose ABPS showed limited efficacy, with minimal reductions in BUN (42.41 ± 5.48 mmol/L), Scr (69.27 ± 12.99 μmol/L), CysC (1.73 ± 0.06 ng/mL), and Kim-1 (98.64 ± 4.47) pg/mL compared to those in the hypoxia group (*p* < 0.001). In contrast, high-dose ABPS markedly improved renal function; BUN decreased by 58.1% (29.72 ± 4.97 mmol/L vs. hypoxia group 70.87 ± 14.3 mmol/L, *p* < 0.05), Scr decreased by 34.5% (48.69 ± 8.05 μmol/L vs. hypoxia group 74.28 ± 13.84 μmol/L, *p* < 0.05), NGAL decreased by 23.6% (103.78 ± 5.24 ng/mL vs. hypoxia group 135.87 ± 12.06 ng/mL, *p* < 0.05), KIM-1 decreased by 29.6% (79.42 ± 3.22 pg/mL vs. hypoxia group 112.94 ± 6.57 pg/mL, *p* < 0.05), and CysC decreased by 32.2% (1.45 ± 0.06 ng/mL vs. hypoxia group 2.14 ± 0.18 ng/mL, *p* < 0.05). The efficacy of ABPH paralleled the positive control dexamethasone (all *p* > 0.05) (Figures 5E–I).

### 3.3 ABPS alleviates hypobaric hypoxia-induced AKI by degrading NETs

**FIGURE 6:**
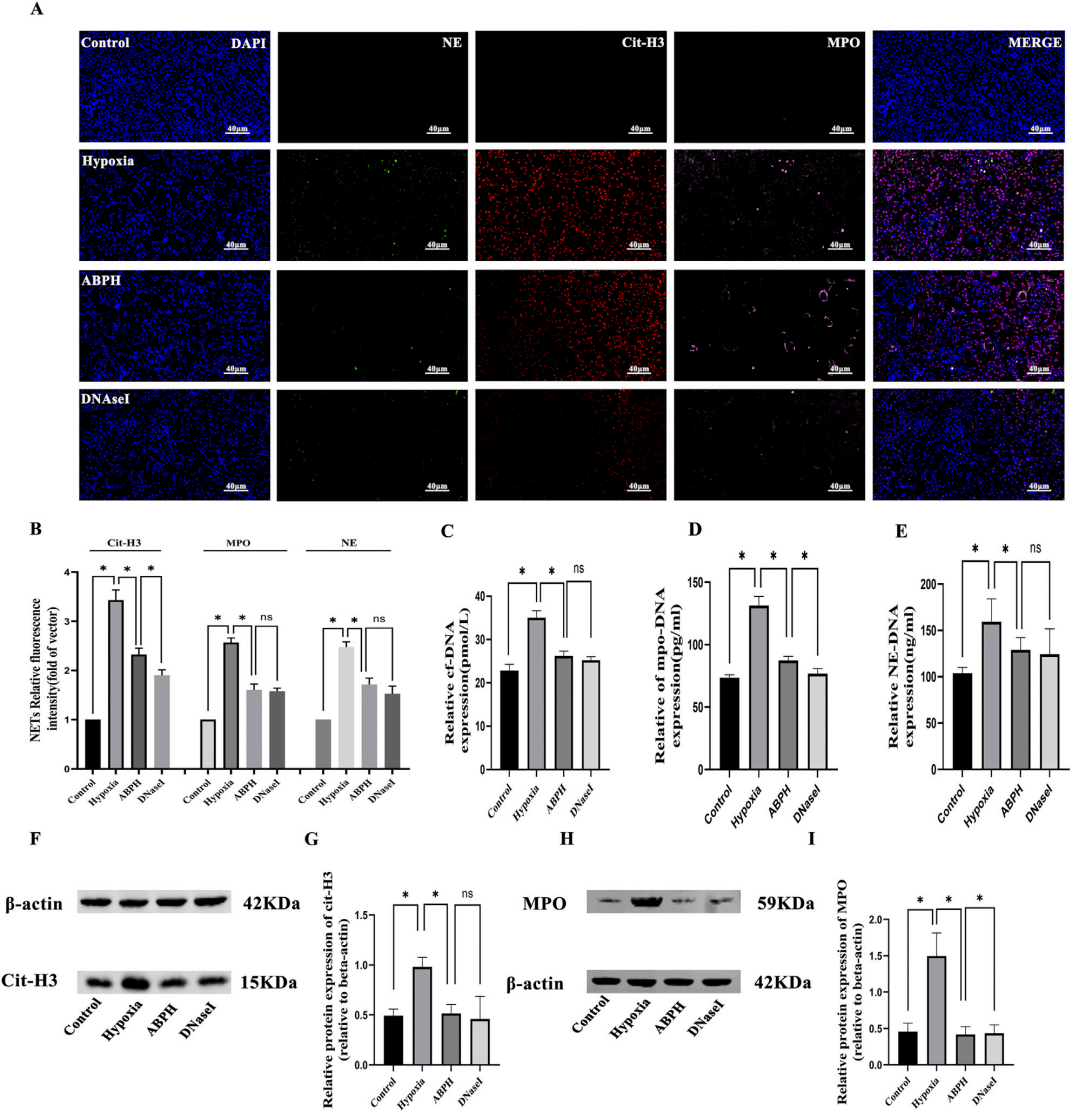
**(A)** Immunofluorescence staining of NETs, neutrophil elastase (NE, green), myeloperoxidase (MPO, pink), and citrullinated histone H3 (cit-H3, red) in renal tissues (×400). Nuclear DNA was counterstained with DAPI (blue). **(B)** Quantitative analysis of NETs, *p < 0.05. **(C–E)** The cf-DNA, MPO-DNA, and NE-DNA levels significantly increased compared to those in the control group, *p < 0.05. **(F–I)** Western blot analysis revealed that cit-H3 and MPO protein expressions significantly increased in the hypoxia group compared to those in the control group, *p < 0.05.

The results of multiple fluorescence immunohistochemistry showed that no NET (MPO, NE, and cit-H3) expression was observed in the renal tissues of the rats in the normal control group. Compared with the normal control group, obvious NET expression was observed in the renal tubular area of the renal tissues of the rats in the hypoxia group, while the expression of NETs in the renal tissues of the ABPH and DNase I groups was significantly reduced by semi-quantitative analysis (*p* < 0.05) (Figures 6A,B).

In the hypoxia model group, significant elevations in NETs were observed compared to those in the control group: cf-DNA (35.05 ± 1.6 pmol/L vs. 22.81 ± 1.47 pmol/L, *p* < 0.001), MPO-DNA (131.3 ± 7.32 pg/mL vs. 73.55 ± 2.21 pg/mL, *p* < 0.001), and NE-DNA (159.5 ± 24.54 ng/mL vs. 103.9 ± 6.22 ng/mL, *p* < 0.001). ABPH intervention effectively reduced these markers (cf-DNA: 26.2 ± 1.09 pmol/L; MPO-DNA: 87.41 ± 3.29 pg/mL; NE-DNA: 128.99 ± 13.25 ng/mL; *p* < 0.001 vs. hypoxia group) compared to the hypoxia group. Moreover, the effects of ABPH intervention were comparable to those observed in the DNase I group (cf-DNA: 25.22 ± 0.81 pmol/L;NE-DNA:124.31 ± 27.52 ng/mL; vs. ABPH, p > 0.05) (Figures 6C–E).

Western blot analysis further confirmed reduced expression of MPO (0.41 ± 0.10 vs. hypoxia group 1.49 ± 0.31, *p* < 0.05) and cit-H3 (0.51 ± 0.08 vs. hypoxia group 0.98 ± 0.09, *p* < 0.05), key indicators of NET formation (Figures 6F–I).

**FIGURE 7:**
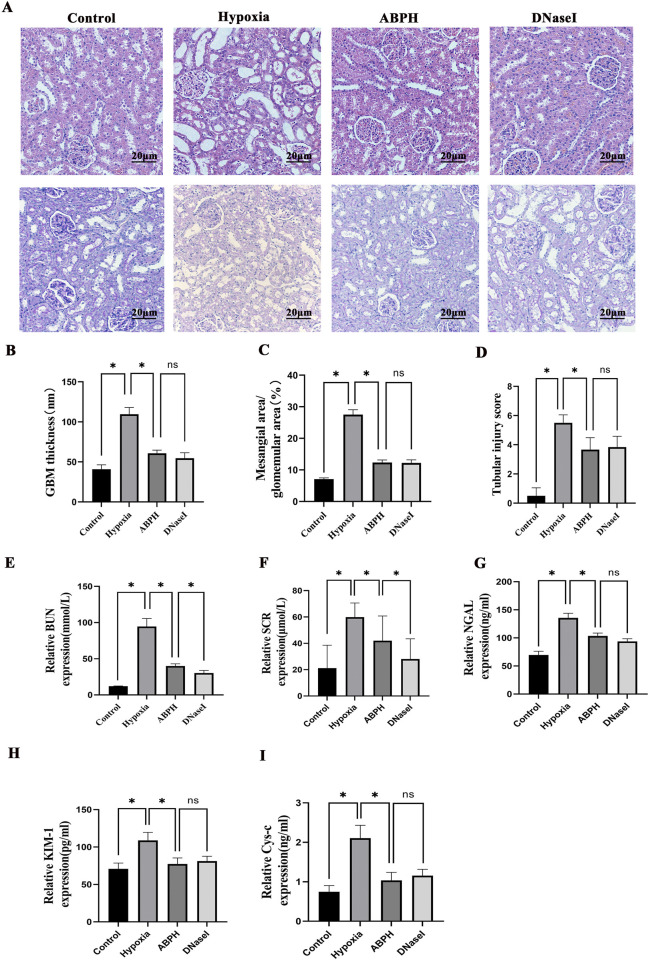
**(A)** H&E staining (×200 magnification) demonstrated renal histopathological alterations. Renal tissues from the control group exhibited no structural damage. In contrast, the hypoxia group displayed significant cortical injury, including marked tubular epithelial cell swelling, cytoplasmic pallor and vacuolization, extensive tubular dilation, flattened or sloughed epithelial cells, and interstitial lymphocyte infiltration. Compared to the hypoxia group, both ABPH and DNase I treatments significantly alleviated these pathological changes. PAS staining (×200 magnification) revealed glomerular morphology. Control group glomeruli showed no abnormalities, whereas the hypoxia group exhibited mesangial cell proliferation and matrix expansion. Notably, ABPH and DNase I treatments markedly reduced mesangial hyperplasia compared to the hypoxia group. **(B–D)** Quantitative scoring analysis of kidney injury. *p < 0.05. **(E–I)** BUN, Scr, NGAL, KIM-1, and CysC levels, *p < 0.001.

Quantitative scoring analysis of kidney histopathological injury via H&E and PAS staining revealed severe tubular necrosis and basement membrane damage in rats of the hypoxia group, which were markedly ameliorated by ABPH, demonstrating reduced inflammatory infiltration and structural preservation (*p* < 0.05) (Figures 7A–D).

Renal dysfunction markers, including BUN (94.62 ± 11.09 mmol/L vs. control group 12.21 ± 0.223 mmol/L, *p* < 0.05), Scr (59.92 ± 10.71 μmol/L vs. control group 21.14 ± 17.39 μmol/L, *p* < 0.05), NGAL (135.62 ± 8.37 ng/mL vs. control group 69.61 ± 6.56 ng/mL, *p* < 0.05), KIM-1 (108.84 ± 10.73 pg/mL vs. control group 70.81 ± 7.79 pg/mL, *p* < 0.05), and CysC (2.11 ± 0.33 ng/mL vs. control group 0.75 ± 0.16 ng/mL, *p* < 0.05), were markedly elevated in the hypoxia group. ABPH treatment significantly restored renal function (BUN: 40.03 ± 2.97 mmol/L; Scr: 41.98 ± 18.77 μmol/L; NGAL: 103.49 ± 5.09 ng/mL; KIM-1:77.34 ± 8.03 pg/mL, CysC: 1.04 ± 0.2 ng/mL, all *p* < 0.001 vs. hypoxia group), achieving effects similar to DNase I (Figures 7E–I).

### 3.4 ABPH suppresses the NLRP3/ASC/caspase-1 pathway by reducing NETs

**FIGURE 8:**
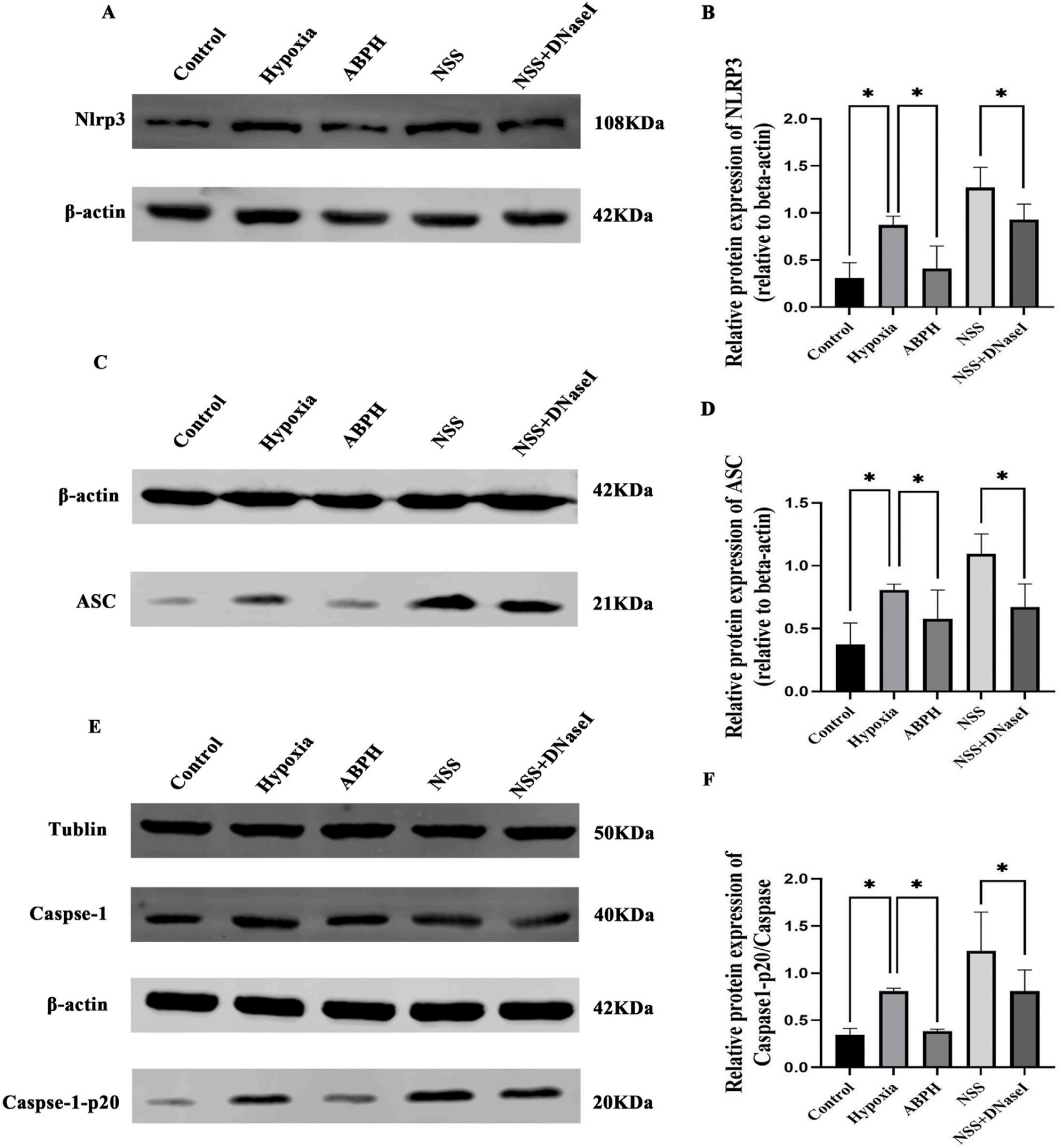
Relative protein expression levels of NLRP3 **(A,B)**, ASC **(C,D)**, caspase-1, and caspase-1-p20 **(E,F)**, *p < 0.05.

In the hypoxia group, protein expressions of NLRP3, ASC, and caspase-1 were significantly elevated compared to those in the controls (NLRP3: 0.87 ± 0.09 vs. 0.30 ± 0.16, *p* < 0.05; ASC: 0.81 ± 0.04 vs. 0.37 ± 0.17, *p* < 0.05). ABPH intervention markedly reduced these levels (NLRP3: 0.41 ± 0.23, 52.9% decrease; ASC: 0.57 ± 0.22, 29.6% decrease). Notably, the NLRP3 agonist NSS exacerbated inflammasome activation (NLRP3: 1.26 ± 0.21; ASC: 1.09 ± 0.15; *p* < 0.05 vs. hypoxia group), while NSS + DNase I partially reversed this effect (NLRP3: 0.92 ± 0.16; ASC: 0.67 ± 0.18; *p* < 0.05 vs. NSS) (Figure 8).

**FIGURE 9:**
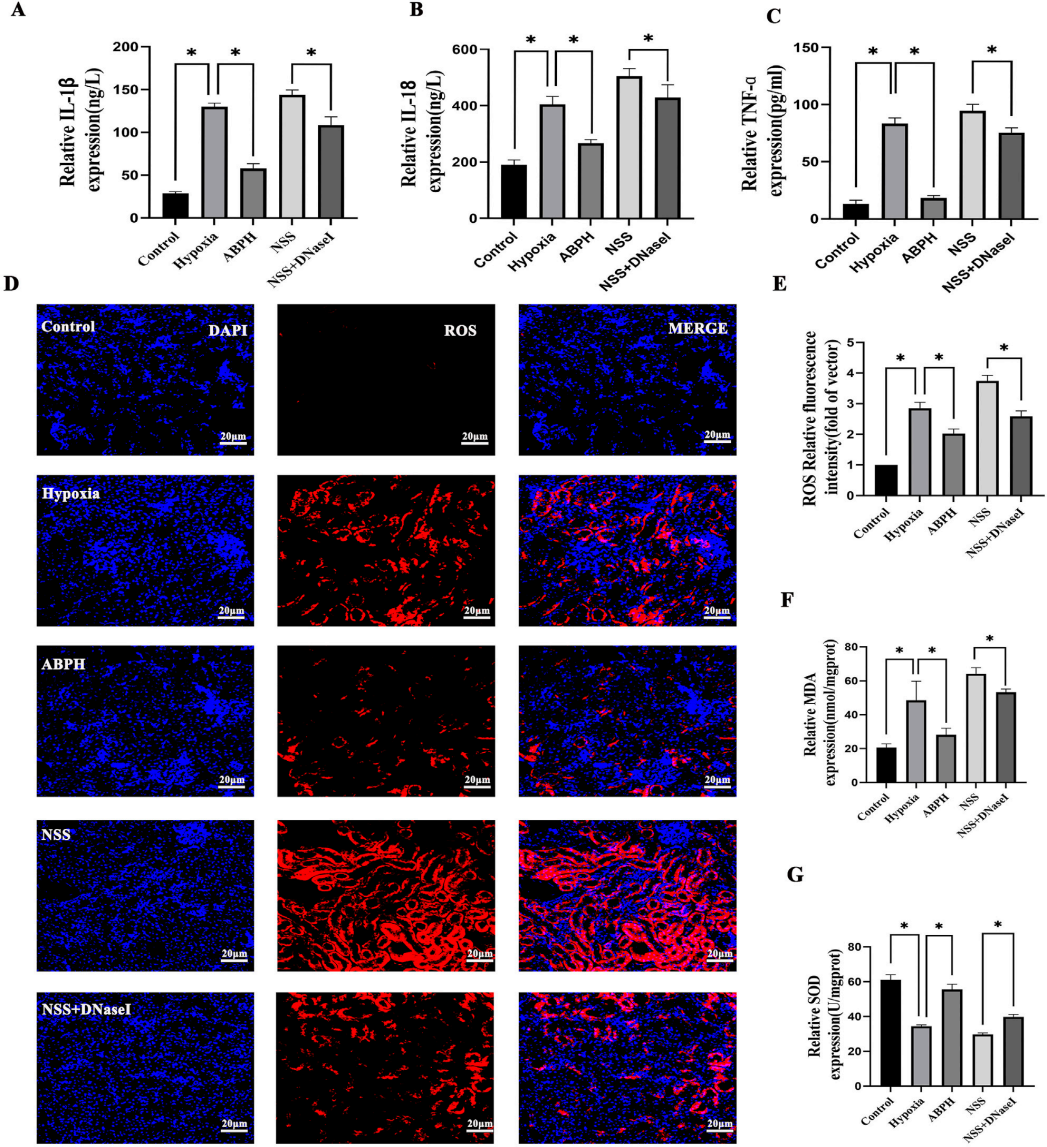
Relative protein expression levels of IL-1β, IL-18, TNF-α **(A–C)**, ROS **(D,E)**, MDA, and SOD **(F,G)**, *p < 0.001.

ABPH attenuates pro-inflammatory cytokines and oxidative stress. Hypobaric hypoxia-induced renal injury triggered a robust release of inflammatory cytokines. ABPH treatment markedly attenuated these responses, reducing IL-1β by 55.4% (57.89 ± 5.74 pg/mL; *p* < 0.001 vs. hypoxia 129.86 ± 4.44 pg/mL), IL-18 by 34% (267.2 ± 13.12 ng/L; p < 0.001 vs. hypoxia 405.05 ± 28.32 ng/L), and TNF-α by 77.9% (18.5 ± 2.1 pg/mL; *p* < 0.001 vs. hypoxia 83.59 ± 4.88 pg/mL) relative to the hypoxia group (Figures 9A–C). Concurrently, oxidative stress markers MDA (48.48 ± 11.36 nmol/mgprot vs. control group 20.59 ± 2.22 nmol/mgprot, *p* < 0.001) and SOD (34.4 ± 0.73 Unit vs. control group 61.03 ± 2.98 U/mgprot, *p* < 0.001) were dysregulated in the hypoxia group. ABPH restored redox balance (MDA: 28.19 ± 3.82 nmol/mgprot 38.5% decrease; SOD: 55.51 ± 3.03 U/mgprot, 45.6% increase; *p* < 0.001 vs. hypoxia group), whereas NSS worsened oxidative damage (MDA: 64.15 ± 3.67 nmol/mgprot; SOD: 29.8 ± 0.75 U/mgprot; *p* < 0.001 vs. hypoxia group). ROS levels demonstrated significant elevation in the hypoxia group. ABPH treatment markedly reduced ROS expression, whereas NSS exacerbated ROS production. The combination of NSS and DNase I partially attenuated ROS levels. These findings suggest that ABPH mitigates oxidative stress and inflammatory responses by NETs (Figures 9D–G).

## 4 Discussion

NETs, a unique immune defense mechanism, have recently been implicated in the pathogenesis of various kidney diseases, particularly AKI (Gupta and Kaplan, 2016). In ischemic renal injury models, infiltrating neutrophils release NETs composed of DNA, cit-H3, and granular proteins (MPO and NE), which directly damage tubular epithelial cells and amplify local inflammatory responses (Nakazawa et al., 2017). Hypobaric hypoxia can induce the NET formation through the NF-κB signaling pathway, resulting in renal injury (Wei et al., 2023). Our study demonstrated a significant elevation of NETs in hypoxic renal injury. These changes correlated positively with the severity of AKI, as indicated by elevated Scr and BUN levels. A clinical study further corroborates these findings, showing that AKI patients exhibit elevated serum levels of cf-DNA and MPO-DNA, which correlate with renal dysfunction (elevated Scr and BUN) and pro-inflammatory cytokines IL-1β and TNF-α release (Tao et al., 2024). Nakazawa et al. (2017) and Desai et al. (2016) demonstrated in a unilateral renal artery clamping model that NETs exacerbate tubular necrosis and remote organ damage via histone- and high-mobility group box 1 (HMGB1)-mediated pathways.

ABPS, a plant indigenous to tropical and subtropical regions of Asia and Africa, exhibits a concentrated distribution across China, Japan, India, and Java. It has been pharmacologically associated with longevity enhancement, musculoskeletal fortification, hepatorenal functional optimization, and hemodynamic facilitation (Chen et al., 2024; Hua and Zhang, 2019). Our results showed that high-dose ABPS demonstrates superior renoprotection over low-dose ABPS, highlighting the importance of dosage optimization. High-dose ABPS restores renal biomarkers and mitigates histopathological damage, comparable to dexamethasone. High-dose ABPS emerges as a potent natural alternative for hypoxic AKI management. Notably, ABPS enhances cisplatin chemosensitivity in breast cancer by disrupting the PD-1/PD-L1 immune checkpoint axis, thereby reversing tumor immunosuppression and improving chemotherapeutic efficacy (Lei et al., 2024). Furthermore, calenduloside, a pentacyclic triterpenoid isolated from *Achyranthes bidentata*, modulates mitochondrial bioenergetics by activating the AMPK–SIRT3 signaling pathway, suggesting its utility in metabolic disorder-related pathologies (Guo et al., 2024). Mechanistic studies reveal that ABPS mitigates chondrocyte endoplasmic reticulum stress via the lncRNA NEAT1/miR-377-3p regulatory axis, offering novel insights into osteoarthritis management (Fu et al., 2022b). Similarly, modified ABPS demonstrates hepatoprotective effects by suppressing apoptosis in hepatocytes under exhaustive exercise-induced oxidative stress, implicating its role in exercise-related hepatic injury prevention (Lin et al., 2010). Importantly, ABPS exhibits neuroprotective potential through selective modulation of NR2A- and NR2B-containing NMDA receptors, which may inform therapeutic strategies for neurodegenerative diseases (Shen et al., 2008). Its robust anti-inflammatory activity is further validated by the inhibition of LPS-induced IL-1β and TNF-α secretion via blockade of NF-κB signaling pathway, positioning ABPS as a promising candidate for inflammatory disorder treatment (Wang et al., 2017).

Our results demonstrated that hypoxic renal injury is characterized by significantly elevated levels of NETs in renal tissues. These findings suggest that ABPS exerts renoprotective effects by either promoting NET degradation or inhibiting their formation. Excessive NET release exacerbates renal injury through direct tubular epithelial cell necrosis and activation of innate immunity via DAMPs. NET components, such as histones and proteases, induce direct cytotoxic effects on renal tubular cells (Nakazawa et al., 2017). NET-derived HMGB1 and other DAMPs trigger inflammatory signaling cascades, amplifying tissue damage (Huang et al., 2015). Our study showed that ABPSs significantly reduced cit-H3, a hallmark of PAD4-mediated histone citrullination, suggesting a potential role in targeting PAD4-dependent NET formation. However, this study cannot precisely confirm whether ABPS degrades NETs or inhibits the formation of NETs, which requires further validation.

Critical role of the NETs–NLRP3 inflammasome axis in hypoxic renal injury: NETs play a dual role in kidney injury, acting as both a defense mechanism against pathogens and a contributor to tissue damage. In hypoxic renal injury, NET components, such as cf-DNA, act as DAMPs that activate NLRP3 inflammasome via the TLR4/MyD88 signaling pathway (Gupta and Kaplan, 2016). Our study revealed a dramatic increase in NLRP3, ASC, and caspase-1 protein expressions in the hypoxia group, which was significantly attenuated by ABPS intervention. This suppression was accompanied by decreased levels of pro-inflammatory cytokines, including IL-1β, IL-18, and TNF-α. These findings align with Nakazawa et al. (2017), who demonstrated that NETs activate NLRP3 through TLR4-dependent signaling in ischemic renal injury, driving inflammatory cascades. Notably, the inhibitory effect of ABPS on the NLRP3 pathway closely correlated with its ability to degrade NETs, suggesting that NETs serve as upstream activators of NLRP3. The comparable NLRP3 suppression observed in the DNase I treatment group further supports the causal chain of “NETs→NLRP3→inflammatory cytokines”.

Emerging evidence underscores the pivotal role of NETs in driving renal pathology through multifaceted mechanisms. Notably, NET-derived DAMPs, including cf-DNA, perpetuate renal inflammation by activating the TLR4/NLRP3 inflammasome axis, which triggers caspase-1-dependent cytokine maturation and establishes a self-amplifying “NETs–inflammasome–tissue injury” loop, a hallmark of progressive kidney damage (Nakazawa et al., 2017). In thrombotic microangiopathies, NETs exacerbate microvascular occlusion by entrapping platelets and erythrocytes, thereby compromising renal perfusion and promoting ischemic injury (Martinod and Wagner, 2014). The inflammatory cascade is further amplified in AKI, where NET-mediated NLRP3 activation induces robust secretion of IL-1β, IL-6, and TNF-α, intensifying renal tubular epithelial cell apoptosis and parenchymal destruction (Nakazawa et al., 2017; Chen et al., 2018). Autoimmune-driven renal pathologies, such as lupus nephritis, are similarly impacted: NETs release cf-DNA and histones that act as autoantigens, stimulating anti-dsDNA antibody production and subsequent immune complex deposition in glomeruli, a key driver of lupus-related nephritogenicity (Hakkim et al., 2010; Zhang et al., 2021). Chronic kidney disease progression is mechanistically linked to NETs through TGF-β1-mediated fibrotic reprogramming. NETs upregulate TGF-β1 signaling in renal fibroblasts, accelerating collagen deposition and pathological extracellular matrix remodeling, which collectively fuel irreversible tubulointerstitial fibrosis (Honda and Kubes, 2018).

Dual mechanisms of ABPSs in modulating oxidative stress: Our study demonstrates that hypoxic renal injury induces mitochondrial dysfunction, leading to excessive ROS generation, which triggers lipid peroxidation and disrupts antioxidant defenses. ABPS intervention effectively attenuated oxidative stress by reducing MDA, restoring SOD activity, and diminishing ROS. This protective effect may arise through two complementary mechanisms: NETs enhance ROS generation through NADPH oxidase activation, leading to mitochondrial dysfunction and RTEC apoptosis (Gupta and Kaplan, 2016; Tang et al., 2015). Additionally, NET degradation by ABPS may indirectly reduce mitochondrial DNA leakage—a potent DAMP that exacerbates oxidative stress by preserving mitochondrial integrity (Desai et al., 2016). This dual action positions ABPS as a multifaceted agent capable of targeting both oxidative stress and inflammation in hypoxic renal injury.

Current therapeutic strategies targeting NETs: DNase I, as a positive control, exhibits biological activity but lacks specific pharmacokinetics. There are several limitations, including short half-life, poor tissue specificity, and immunogenicity risks. DNase I possesses clear target specificity and can directly degrade NETs. However, DNase I is not stable *in vivo* and can only be administered via the intravenous route. ABPS demonstrated comparable efficacy to DNase I in reducing NETs and inflammatory cytokines while offering additional benefits in ameliorating oxidative stress, underscoring its multi-target advantage. ABPS has a clear pharmacokinetic profile and can be administered via intravenous and oral routes. Although our study confirmed that it can reduce the formation of NETs, whether it directly targets and degrades NETs still requires further research. Additionally, this study cannot establish the dose equivalence between ABPS and DNase I. In summary, DNase I rapidly degrades extracellular DNA but lacks sustained bioavailability, whereas the ABPS structure may enhance tissue retention and multi-target effects.

Furthermore, as a natural botanical drug, ABPS exhibits low toxicity and high biocompatibility. However, its precise molecular targets—such as the direct inhibition of PAD4 or TLR4—require further validation through genetic knockout or pharmacological inhibitor experiments. All the results showed that ABPS is a promising experimental agent.

There are several limitations to this study. The study does not differentiate whether ABPS actively promotes the breakdown of existing NETs, inhibits their formation, or both. Although the cit-H3 level was measured, no experiments were conducted to assess PAD4 expression or enzymatic activity, which is essential for NETosis. Further studies should include the PAD4-knockout models to clarify the proposed NET-inhibitory effect. Additionally, the immunofluorescence data used to visualize NETs do not include negative controls such as isotype-matched antibodies or DNase I-treated sections. Although this study further conducted WB verification of NET-related component proteins MPO and cit-H3, these are essential to confirm the specificity of observed signals, particularly in multiplex imaging, where overlap between DNA and cytoplasmic proteins can lead to false positives. Furthermore, systemic hypoxia may be a confounding factor in the hypobaric hypoxia-induced acute renal injury model; therefore, renal function indicators, cf-DNA and MPO-DNA were measured from serum. Finally, for the ELISA experiments, the blank control values were determined on the same plate as the standard curve and subtracted from corresponding sample measurements. The concentrations of MDA and SOD were determined using the BCA kit standard curve. Different plates have their own background noises due to different incubation times and conditions, which represents one of the technical limitations.

In conclusion, this study provides the first evidence that ABPS alleviates hypoxic renal injury by reducing NET formation, blocking NLRP3 inflammasome activation, and synergistically modulating oxidative stress. ABPS shows potential as a multi-target, low-toxicity candidate for renal protection, although further pharmacokinetic and safety studies are needed before clinical application.

## Data Availability

The original contributions presented in the study are included in the article/supplementary material, further inquiries can be directed to the corresponding authors.
